# Causal role of immune cells in uveitis: a Mendelian randomization study

**DOI:** 10.3389/fmed.2024.1445775

**Published:** 2024-07-31

**Authors:** Jianping Pu, Zhuanghong Zhao, Yanping Duan, Jun Lu, Yuchen Yao, Yuxin Wen, Yanxun Li, Yu Zhang, Fengyu Ye

**Affiliations:** ^1^Department of Ophthalmology, Anning First People's Hospital Affiliated to Kunming University of Science and Technology, Kunming, Yunnan, China; ^2^Department of Pathology, Kunming Maternal and Children Hospital, Kunming, Yunnan, China; ^3^School of Basic Medical Sciences, Kunming Medical University, Kunming, Yunnan, China; ^4^Department of Pathology, The First Affiliated Hospital of Kunming Medical University, Kunming, Yunnan, China

**Keywords:** immune cells, Mendelian randomization, uveitis, genome wide association study, causal relationship

## Abstract

**Background:**

Uveitis refers to a group inflammation affecting the uvea, retina, retinal blood vessels as well as vitreous body, which is one of the common causes of blindness. There is growing evidence linking different types of immune cells to uveitis, although it remains uncertain if these associations imply causal relationships. Recent advancements in high-density genetic markers like SNPs or CNVs for genotyping, along with the progress in genome-wide association studies (GWAS) technologies, have improved our understanding of the immunological mechanisms involved in ocular diseases. Therefore, our objective was to investigate the potential causal link between immune cells and uveitis using a Mendelian randomization study.

**Methods:**

The exposure and outcome GWAS data for this study were sourced from an open-access database (https://gwas.mrcieu.ac.uk/). Two-sample MR analysis was utilized to evaluate the causal relationship between 731 immune cell features and uveitis. Various MR methods were employed to reduce bias and obtain dependable estimates of the causal link between the immune cell variables and the outcomes. Instrumental variable selection criteria were carefully chosen to enhance the accuracy and efficacy of the causal relationship between different immune cell types and the risk of uveitis.

**Results:**

Using two-sample MR, IVW modeling showed that GAD had significant effect on immunophenotypes. CD3 levels on CD45RA− CD4+ T cells (OR = 1.087, 95%CI = 1.029 ~ 1.147, *p* = 0.003) and CD3 levels on CM CD4+ T cells (OR = 1.086, 95%CI = 1.033 ~ 1.141, *p* = 0.001) were found to be elevated in cases of uveitis. HLA DR levels in CD14− CD16+ monocyte cells (OR = 0.735, 95% CI = 0.635 ~ 0.850, *p* < 0.001) and HLA DR levels in NK cells (OR = 0.910, 95% CI = 0.851 ~ 0.972, *p* = 0.005) were observed to be reduced in individuals with uveitis. Furthermore, Two cells were identified to be significantly associated with uveitis risk: HLA DR on in NK cells (OR = 0.938, 95%CI = 0.899 ~ 0.979, *p* = 0.003), HLA DR on CD14− CD16+ monocytes (OR = 0.924, 95%CI = 0.878 ~ 0.972, *p* = 0.002).

**Conclusion:**

This study highlights the intricate relationship between immune cells and generalized anxiety disorder using genetic methods, offering valuable insights for future clinical investigations.

## 1 Introduction

Uveitis is characterized by inflammation that impacts various parts of the eye, such as the uvea, retina, retinal blood vessels, and vitreous body (1, 2). This inflammatory condition can also affect organs beyond the eye, including the skin, joints, and blood vessels. Skin manifestations of uveitis may include erythematous rashes, papules, or nodules (3). Furthermore, uveitis can lead to vasculitis, an inflammation of blood vessels, potentially causing vascular damage and compromising blood supply to different organs (4). The underlying mechanism of uveitis involves an abnormal immune response, where immune cells play a pivotal role in the autoimmune destruction of the uvea and other affected organs (5). In developing countries, uveitis and its complications account for approximately 25% of cases of irreversible blindness (6–8). Uveitis is a prevalent condition among individuals in the working age group, with significant implications for their quality of life and substantial socioeconomic consequences (9).

Extensive research has uncovered the complex interplay between uveitis and the immune system. Uveitis is an autoimmune disease affecting the central nervous system, with abnormalities in different immune cells playing a key role in the autoimmune destruction of the uvea (10, 11). CD4+ T cells, including regulatory (Treg) and effector (such as T helper (Th)-1 and Th17 cells), play a crucial role in the development of uveitis and its well-known animal model, experimental autoimmune uveitis (12, 13). There is growing evidence supporting a strong connection between activated γδ T cells and the onset of autoimmune uveitis. Upon activation, γδ T cells have the capacity to secrete multiple cytokines such as interleukin IL-17, tumor necrosis factor-α (TNF-α), and interferon-γ (IFN-γ), thereby playing a crucial role in modulating the immune response and contributing significantly to the pathogenesis of autoimmune uveitis (14–16). These studies suggest great potential for investigating immune modulation as a means of controlling uveitis. These findings have made a significant contribution to enhancing our understanding of the pathogenesis of uveitis, offering valuable insights for potential future treatments and preventive measures. Nonetheless, current research outcomes regarding the relationship between immune cells and uveitis have displayed inconsistencies, potentially stemming from restricted sample sizes, flawed study methodologies, and confounding variables not yet explored in existing research.

Mendelian randomization (MR) analysis is a powerful technique that leverages genetic variations as instrumental variables (IVs) to investigate potential causal relationships between exposures and outcomes (17–19). This minimizes the impact of confounding factors on causal estimation. In this study, we conducted Mendelian randomization (MR) analysis using published genome-wide association study (GWAS) summary data to investigate the potential causal relationship between immune cell traits and uveitis.

## 2 Materials and methods

### 2.1 Study design

**Figure 1:**
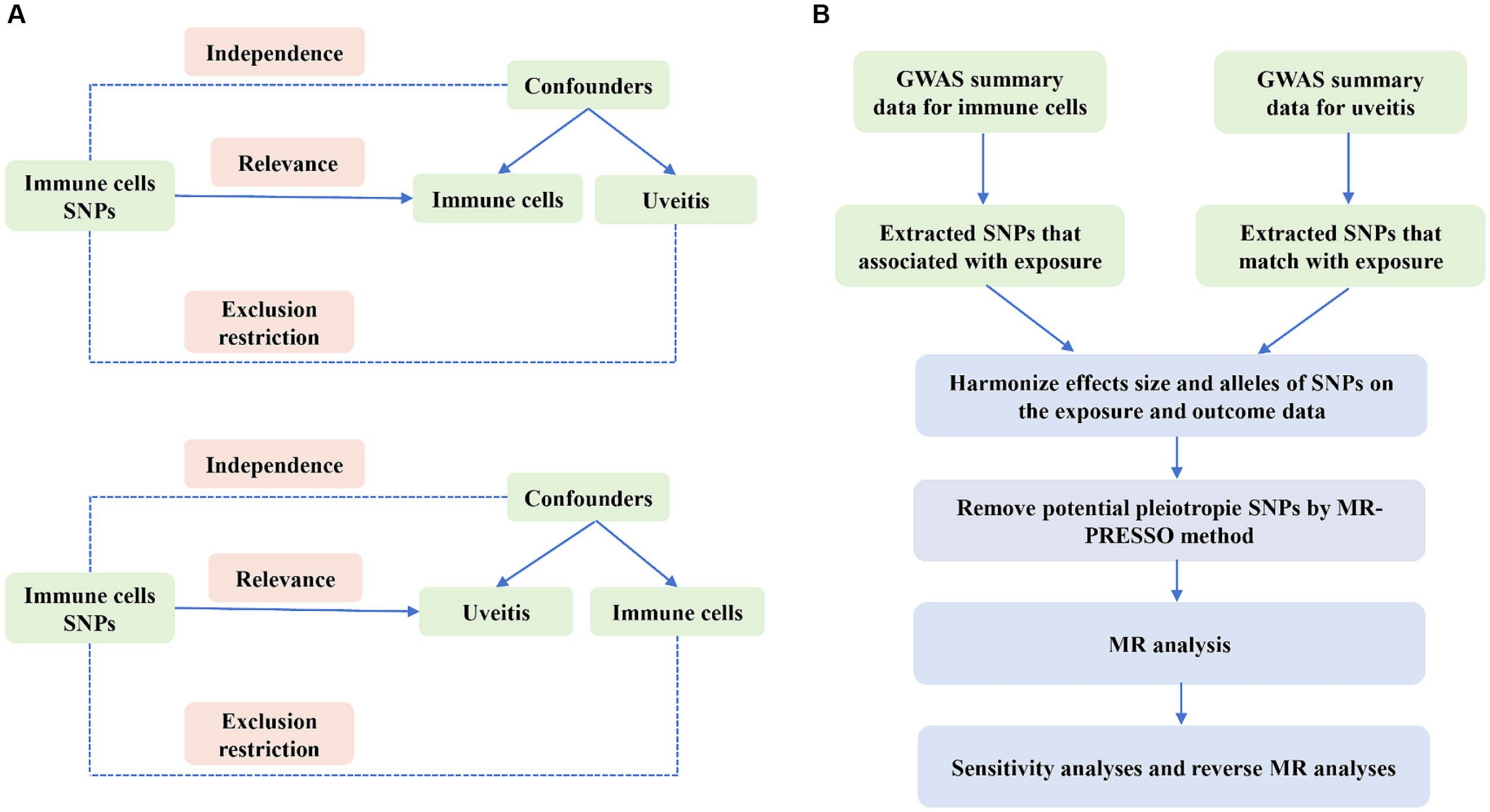
Overview of the overall MR design. **(A)** The diagram of MR assumption. **(B)** The diagram of MR analysis processing.

We examined the causal relationship between 731 immune cell characteristics and uveitis through the use of two-sample Mendelian randomization (MR) techniques. MR is a statistical method that utilizes genetic variants as instrumental variables (IVs) to infer causality. In order to ensure the validity of causal inferences, instrumental variables (IVs) must meet three essential presumptions: (1) the exposure is directly associated with genetic variation, suggesting that genetic variation can affect immune cell characteristics and potentially impact uveitis. (2) There is no genetic correlation between the exposure and outcome that could act as a confounding variable; genetic variation cannot serve as a valid IV if it is connected to such confounders. (3) There is no genetic influence on the outcome through pathways that are unrelated to the exposure. An outline of the research design is depicted in Figure 1.

### 2.2 Sources of immunity-spanning GWAS data

Summary statistics for all immunological features were acquired from the GWAS catalog (ranging from registration number GCST0001391 to GCST0002121) (20). GWAS involved 3,757 nonoverlapping European individuals. A high-density array, based on a reference panel of Sardinian sequences, estimated approximately 22 million SNPs and tested for correlation after controlling for covariates such as age, age^2, and sex. A total of 731 immunophenotypes were examined, including relative cell counts (RC) (192), morphologic parameters (MP) (32), absolute cell counts (AC) (118), and median fluorescence intensities (MFI) representing surface antigen levels (389). The MP features included CDC and TBNK panels, while MFI, RC, and AC features included B cells, CDC, T cell maturation stage, myeloid cells, monocytes, and TBNK (T cells, B cells, natural killer proteins).

### 2.3 Data sources from the genome-wide association study for uveitis

The uveitis GWAS data we used in this study were obtained from the European Bioinformatics Institute (EBI). These encompass a range of uveitis types, including but not limited to: Acute anterior uveitis (AAU), the most prevalent form primarily impacting the anterior uvea and characterized by distinct genetic markers. Behçet’s disease-associated uveitis (BU), a subtype linked to Behçet’s disease, which manifests across multiple ocular regions and involves a intricate interplay of immune and genetic factors. Vogt-Koyanagi-Harada disease (VKH), an autoimmune condition predominantly affecting the eyes but potentially impacting other bodily systems, with specific HLA associations. The study included 480,778 European individuals (2,616 cases and 478,162 controls), and a total of 24,194,599 single nucleotide polymorphisms (SNPs) were analyzed after quality control and filtering. The data can be accessed at: https://gwas.mrcieu.ac.uk/datasets/ebi-a-GCST90018938/.

### 2.4 Selection of instrumental variables

A set of instrumental variables (IVs) (version v1.90) was utilized to modify the SNPs, with a chain disequilibrium [LD] r2 threshold of less than 0.1 at a distance of 500 kb. The LD r2 was calculated using the 1,000 Genomes Project as a reference panel. The new Uveitis significance threshold is set at 5 × 10^−8^. The F statistic was computed to evaluate IV strength and address any weak instrumental biases. The length of the IV for immunophenotypes varied from 3 to 1,643, explaining an average of 0.137% (ranging from 0.009 to 0.995%) of the variance in the relevant immune characteristics.

### 2.5 Statistical analysis

The R version 4.3.1 program^1^ was consistently used in our studies. We employed median-based weighted analyses, pattern-based weighted analyses, and inverse variance weighted analyses (IVW) (21) with the ‘Mendelian Randomization’ software (version 0.4.3) (22) to explore the causal relationship between the 731 immunophenotypes and Uveitis. We assessed instrumental heterogeneity between variables using Cochran’s Q statistic, *p*-value (IV), and the MR-Egger method to detect horizontal multidimensionality. Additionally, we applied the MR-PRESSO technique within the MR-PRESSO package to identify and remove horizontal multidirectional entropy outliers that could bias estimation results (23). After removing these SNPs, we re-ran the IVW analysis. We also searched for SNPs with suggestive associations (*p* < 10^−5^) on the Phenoscanner V2 website.^2^ Finally, we used funnel plots and scatter plots, which showed minimal impact of outliers on the data and a strong association with low heterogeneity.

In this reverse Mendelian randomization analysis, we investigated the potential for reverse causality and explored the impact of uveitis on immune cell characteristics using the same MR methodology. Uveitis was considered as the exposure variable in this reverse MR study, with various immune cell traits being considered as the outcomes.

## 3 Results

### 3.1 Examination of the causal relation of uveitis onset on immunophenotypes

In our investigation of uveitis and its potential impact on immunophenotypes, we employed the Inverse Variance Weighted (IVW) method as the primary analysis in a two-sample Mendelian randomization (MR) study. Our findings revealed four suggestive immunophenotypes: two within the T cell panel, one within the monocyte panel, and one within the NK cell panel.

**Figure 2:**
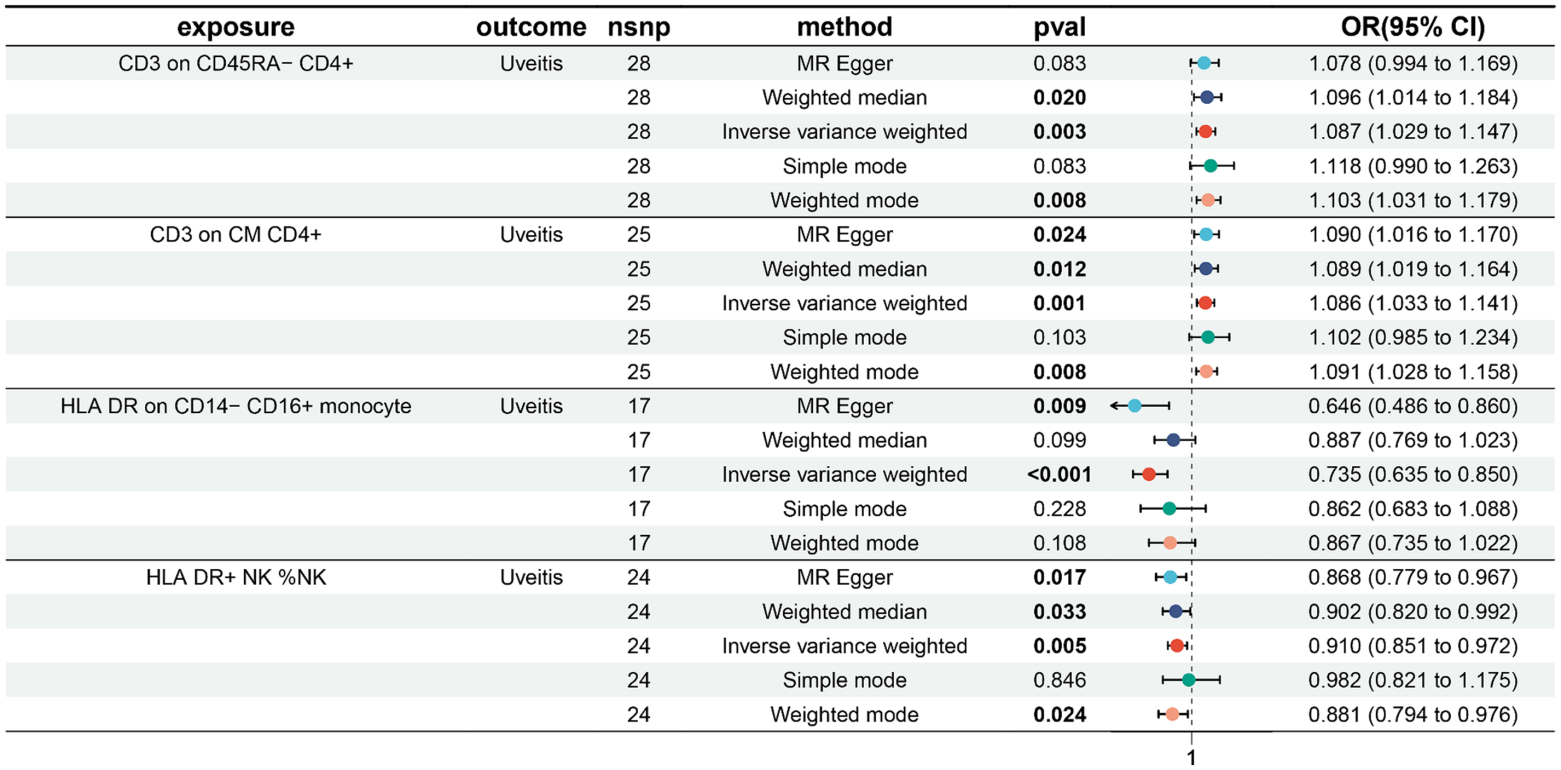
Forest plots showed the causal relations between uveitis and immune cell traits.

Our research indicates that the pathogenesis of uveitis leads to an increase in CD3 levels in CD45RA− CD4+ cells (IVW: odds ratio (OR) 1.087, 95% confidence interval (CI) 1.029–1.147; *p* = 0.003, Figure 2; Supplementary Table S1). Additionally, uveitis pathogenesis is associated with an increase in CD3 levels in CM CD4+ cells (IVW: odds ratio (OR) 1.086, 95% confidence interval (CI) 1.033–1.141; *p* = 0.001, Figure 2; Supplementary Table S2), a decrease in HLA DR levels in CD14− CD16+ monocyte cells (IVW: odds ratio (OR) 0.735, 95% confidence interval (CI) 0.635–0.850; *p* < 0.001, Figure 2; Supplementary Table S3), and a decrease in HLA DR levels in NK cells (IVW: odds ratio (OR) 0.910, 95% confidence interval (CI) 0.851–0.972; *p* = 0.005, Figure 2; Supplementary Table S4).

**Figure 3:**
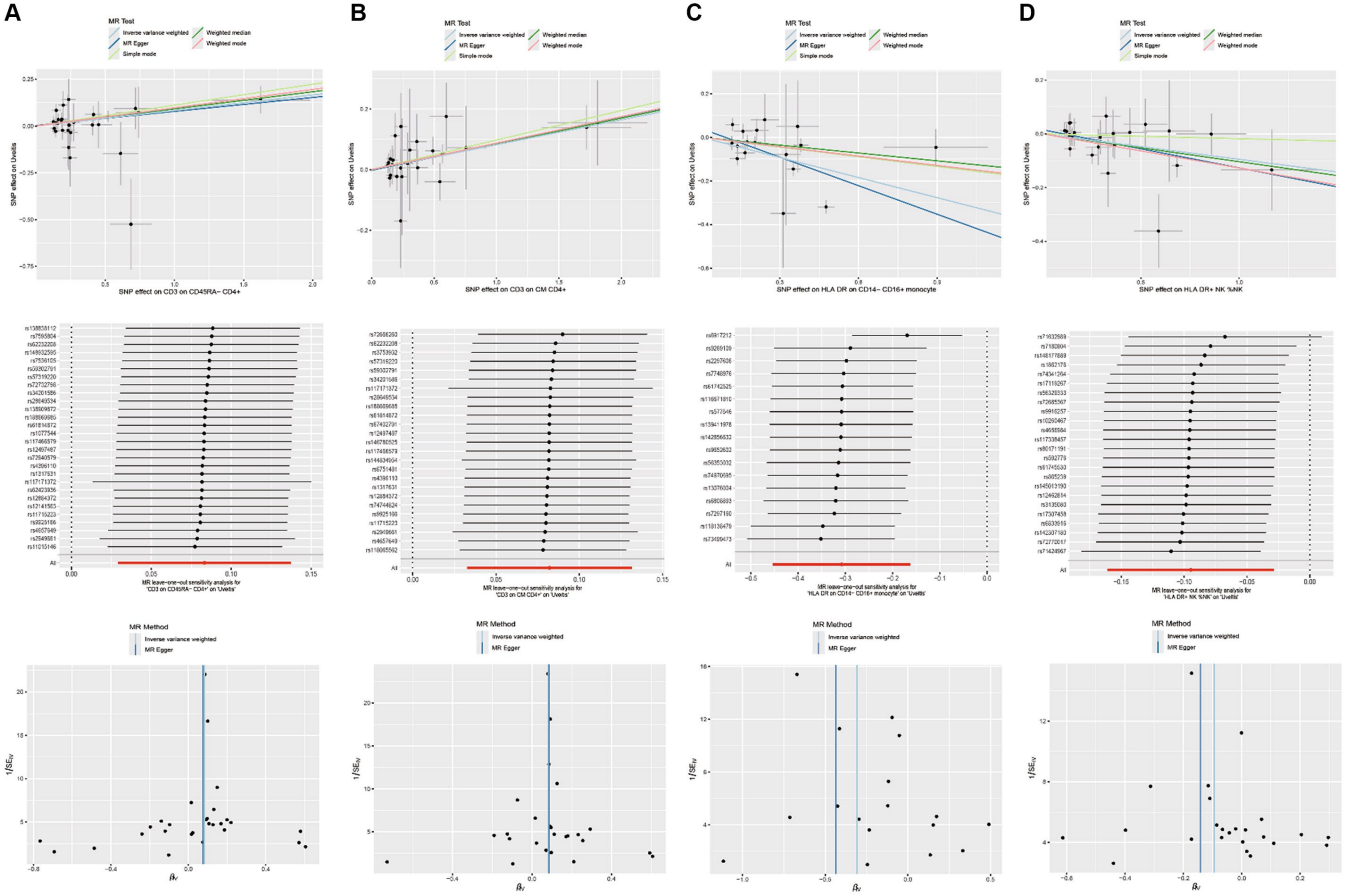
Scatterplots, leave-one-out analysis, and funnel plots showed the causal associations between immune cell traits and uveitis by using different methods. **(A)** CD3 on CD45RA− CD4+ cells. **(B)** CD3 on CM CD4+ cells. **(C)** HLA DR on CD14− CD16+ monocyte cells. **(D)** HLA DR on NK cells.

The results from the other three methods and sensitivity analyses further support the strength of the observed causal associations (Supplementary Tables S1–S4). Specifically, the MR-Egger intercept and MR-PRESSO global tests have ruled out the possibility of horizontal pleiotropy. Additionally, scatterplots, leave-one-out analysis, and funnel plots have demonstrated the stability of the results (Figure 3).

### 3.2 Examination of the causal relation of immunophenotypes on uveitis

In our study on immunophenotypes and their potential impact on uveitis, we identified two immunophenotypes that exhibit inhibitory effects against uveitis: HLA DR levels in NK cells and HLA DR on CD14− CD16+ monocyte.

**Figure 4:**
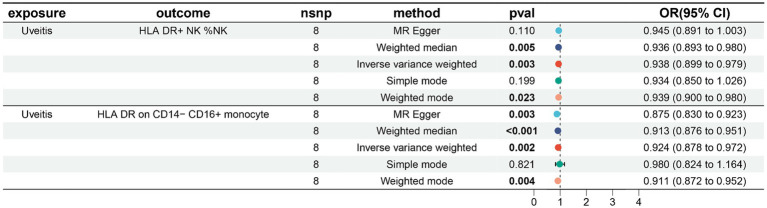
Forest plots showed the causal associations between immune cell traits and uveitis by using different methods.

In particular, the ratio of HLA DR levels in NK cells to uveitis risk (OR) was assessed with the IVW method and was 0.938 (95% confidence interval (CI) 0.899–0.979; *p* = 0.003, Figure 4; Supplementary Table S5), and the ratio of HLA DR on CD14− CD16+ monocyte to uveitis risk (OR) was assessed with the IVW method and was 0.924 (95% confidence interval (CI) 0.878–0.972; *p* = 0.003, Figure 4; Supplementary Table S6).

Three other methods yielded similar results: weighted mode (OR = 2.718, 95% CI = 2.090 ~ 3.535, *p* < 0.001); weighted median (OR = 2.718, 95% CI = 2.309 ~ 3.200, *p* < 0.001); and MR-Egger (OR = 2.718, 95% CI = 2.268 ~ 3.258, *p* < 0.001).

**Figure 5:**
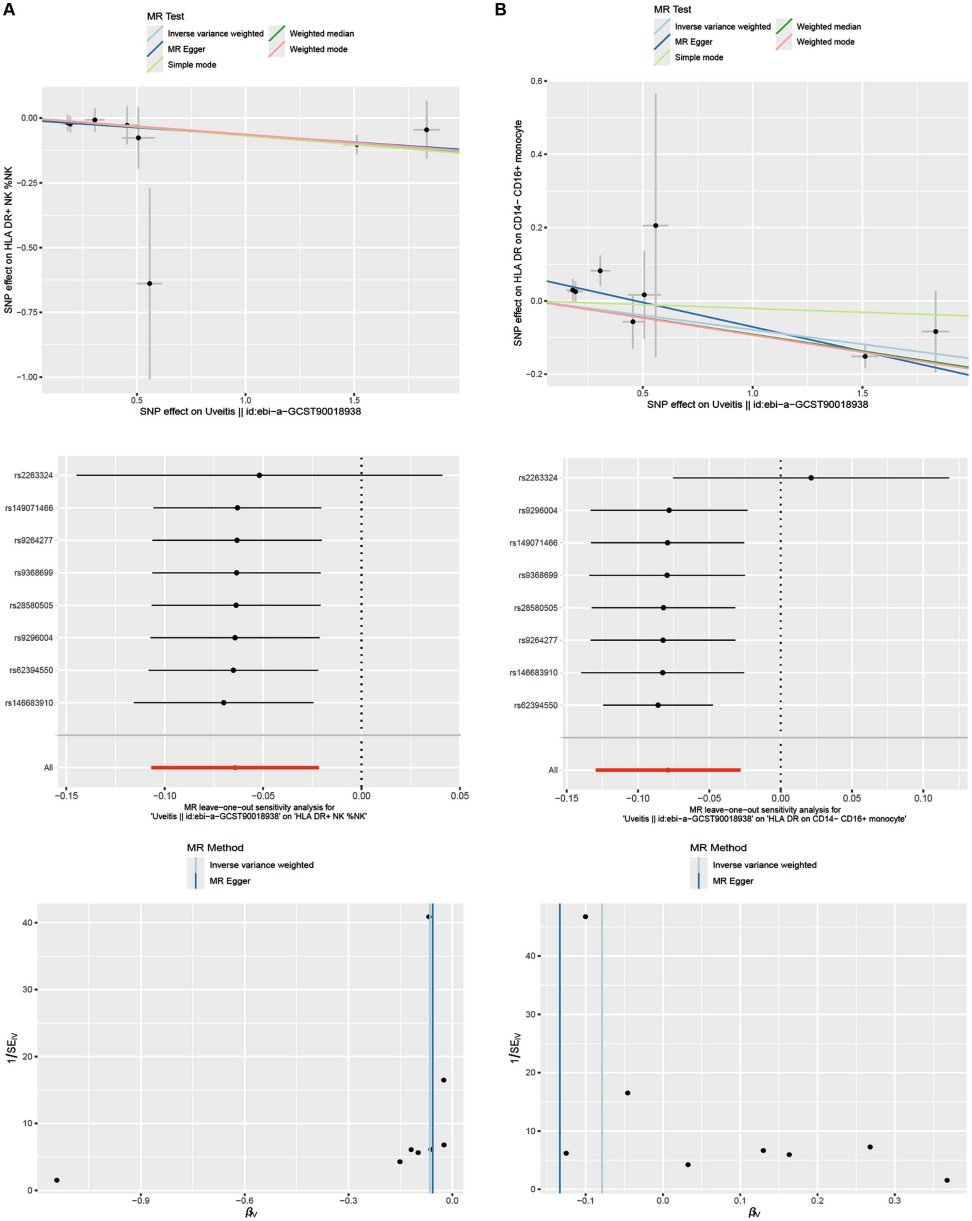
Scatterplots, leave-one-out analysis, and funnel plots showed the causal associations between uveitis and immune cell traits by using different methods. **(A)** HLA DR on NK cells. **(B)** HLA DR on CD14− CD16+ monocyte cells.

In addition, both the MR-Egger intercept and MR-PRESSO global tests dismissed the presence of horizontal pleiotropy. Sensitivity analyses were conducted to confirm the robustness of the observed causal relationships (Figure 4; Supplementary Tables S5–S6). The stability of the data was also illustrated through scatterplots, leave-one-out analysis, and funnel plots (Figure 5).

## 4 Discussion

Based on an extensive analysis of genetic data, our research delved into the causal links between 731 immune cell characteristics and uveitis. This study stands out as the sole Mendelian randomization (MR) investigation to investigate the causal association between various immune traits and uveitis. The study included four immune trait categories (MFI, RC, AC, and MP). Through rigorous inclusion criteria and thorough sensitivity analysis, we uncovered potential causal connections between three distinct immune cell types and uveitis.

Our research indicates that an increased CD3 on CM CD4+ T cells and CD3 on CD45RA− CD4+ T cells percentage is associated with an increased risk of uveitis. CD4+ T cells have a wide range of functions within the immune system. Vaccinologists frequently highlight their role in promoting class switching, somatic hypermutation, and memory differentiation in B cells (24, 25). Additionally, CD4+ T cells act as important effectors, killers, and potent communicators. They play a crucial role in regulating tissue homeostasis, wound healing, and signaling the presence of microbial invasion (26, 27). CD3 on CM CD4+ T cells have the ability to differentiate into various T cell subpopulations including Th1, Th2, Th9, Th17, Th22, and follicular Th cells (Tfh). Each of these subpopulations serves a distinct and vital function in both the immune system and the development of uveitis (28).

Our study found that CD3 on CM CD4+ T cells and CD3 on CD45RA- CD4+ T cells interacted with uveitis, thus CD3 on CM CD4+ T cells and CD3 on CD45RA- CD4+ T cells play a pivotal role in the development of uveitis. HLA-DR belongs to the classic HLA Class I antigen, which is associated with a variety of autoimmune diseases, including uveitis. The link between HLA-DR (human leukocyte antigen-DR) and autoimmune disease has attracted considerable attention. The HLA gene is considered the most significant genetic risk factor in the development of rheumatoid arthritis, with the HLA-DR allele being particularly crucial as it encodes the HLA-DR beta chain. Within the HLA-DRβ, there is a specific 5-amino acid sequence motif known as the shared epitope, which has a strong association with susceptibility to rheumatoid arthritis (29, 30). The onset and progression of Parkinson’s disease have been found to be associated with HLA-DR (31). Research suggests that the level of HLA-DR expression in patient monocytes is linked to improved cognitive function, semantic fluency, and motor function. However, studies using animal models of Parkinson’s disease, which involved acute toxins, protein injections, and pathological abnormalities, have shown that increased HLA-DR expression can have detrimental effects (32, 33). According to earlier research, HLA-DR is not only highly expressed in uveitis, but also HLA-DR and HLA Class I antigen may be a significant factor in the immunological abnormalities connected to uveitis (34, 35). The discovery of HLA-DR and HLA Class I antigens may have a significant impact on uveitis, potentially leading to advancements in its prevention, treatment, and the identification of new therapeutic targets.

In this study, we utilized both standard Mendelian randomization (MR) analysis and reverse Mendelian randomization (reverse MR) analysis to investigate the causal link between immune cell characteristics and uveitis. Both analytical approaches revealed a significant correlation between immune cell features and uveitis. While standard MR analysis identified elevated CD3 levels in CM CD4+ T cells and CD45RA- CD4+ T cells as risk factors for uveitis, reverse MR analysis confirmed this causal relationship with slightly varying odds ratios and confidence intervals. Moreover, reverse MR analysis uncovered the reverse causality of uveitis on immune cell characteristics, enhancing our understanding of the bidirectional role of immune cells in uveitis pathogenesis. The consistent findings from both methods bolster our conclusions on the involvement of immune cells in uveitis mechanisms. The convergence of results from different analytical methods enhances the robustness of our findings. Discrepancies in results may hint at biological distinctions or methodological sensitivities that warrant further investigation and validation in future research. By integrating standard MR analysis and reverse MR analysis, we obtained a more holistic view of the intricate interplay between immune cell profiles and uveitis, offering valuable insights for future clinical investigations and therapeutic approaches.

This study utilized a two-sample Mendelian randomization analysis with data from a substantial genomic research cohort comprising around 342,243 individuals, ensuring high statistical efficiency. The study’s conclusions rely on genetic instrumental variables, and causal inference was conducted using various MR analysis methods. The findings are robust and unaffected by horizontal pleiotropy and other confounding factors.

This study does have several drawbacks. Firstly, the majority of participants in the GWAS summary data utilized in our research are of European descent, potentially limiting the applicability of our findings to other racial groups. Therefore, caution should be exercised when extrapolating these results to populations with different genetic backgrounds. Secondly, thoroughly assessing horizontal pleiotropy remains challenging despite multiple sensitivity analyses. Moreover, the lack of individual-specific data hindered a more detailed, stratified examination of the population. In conclusion, while randomized controlled trials (RCTs) could theoretically minimize the influence of confounding variables and provide stronger in conclusion, while randomized controlled trials (RCTs) could theoretically minimize the influence of confounding variables and provide stronger evidence for causality, we recognize that in the context of uveitis, conducting RCTs may pose significant ethical and practical challenges. Therefore, alternative approaches such as longitudinal observational studies or well-designed cohort studies may be more feasible and still provide valuable insights into the causal relationships between immune cell traits and uveitis. More importantly, neutrophils have been identified as significant contributors to the pathogenesis and outcomes of uveitis (36). By investigating the specific role of neutrophils in uveitis, researchers have the potential to discover innovative strategies to regulate their activation, recruitment, or effector functions, ultimately paving the way for targeted therapies. Although our study did not directly investigate the role of neutrophils, future research should carefully examine their impact in the context of uveitis, as a thorough understanding of their involvement could offer a more comprehensive insight into the disease mechanisms.

## Data availability statement

The original contributions presented in the study are included in the article/Supplementary materials, further inquiries can be directed to the corresponding authors.

## Author contributions

JP: Writing – original draft, Writing – review & editing. ZZ: Data curation, Writing – review & editing. YD: Writing – review & editing, Software. JL: Writing – review & editing, Methodology. YY: Writing – review & editing, Methodology. YW: Supervision, Writing – review & editing. YL: Writing – review & editing, Methodology. YZ: Writing – original draft, Writing – review & editing. FY: Writing – original draft, Writing – review & editing.

## Footnotes

^1^ http://www.Rproject.org

^2^ http://www.phenoscanner.medschl.cam.ac.uk/
